# Hepatic caveolin‐1 is enhanced in *Cyp27a1/ApoE* double knockout mice

**DOI:** 10.1002/2211-5463.12123

**Published:** 2016-09-26

**Authors:** Line Zurkinden, Yosef T. Mansour, Beatrice Rohrbach, Bruno Vogt, Hiten D. Mistry, Geneviève Escher

**Affiliations:** ^1^ Department of Nephrology, Hypertension, Clinical Pharmacology and Clinical ResearchUniversity of BernSwitzerland; ^2^ Division of Women's HealthKing's College LondonWomen's Health Academic CentreUK; ^3^ Division of Child Health, Obstetrics & GynaecologySchool of MedicineUniversity of NottinghamUK

**Keywords:** atherosclerosis, cholesterol efflux, cholesterol metabolism, liver

## Abstract

Sterol 27‐hydroxylase (CYP27A1) is involved in bile acid synthesis and cholesterol homoeostasis. *Cyp27a1* ^(−/−)^/*Apolipoprotein E* ^(−/−)^ double knockout mice (DKO) fed a western diet failed to develop atherosclerosis. Caveolin‐1 (CAV‐1), the main component of caveolae, is associated with lipid homoeostasis and has regulatory roles in vascular diseases. We hypothesized that liver CAV‐1 would contribute to the athero‐protective mechanism in DKO mice. *Cyp27a1* ^(+/+)^/*ApoE* ^(−/−)^ (ApoE KO), *Cyp27a1* ^(+/−)^/*ApoE* ^(−/−)^ (het), and DKO mice were fed a western diet for 2 months. Atherosclerotic plaque and CAV‐1 protein were quantified in aortas. Hepatic *Cav‐1* mRNA was assessed using qPCR, CAV‐1 protein by immunohistochemistry and western blotting. Total hepatic and plasma cholesterol was measured using chemiluminescence. Cholesterol efflux was performed in RAW264.7 cells, using mice plasma as acceptor. CAV‐1 protein expression in aortas was increased in endothelial cells of DKO mice and negatively correlated with plaque surface (*P* < 0.05). In the liver, both CAV‐1 protein and mRNA expression doubled in DKO, compared to ApoE KO and het mice (*P* < 0.001 for both) and was negatively correlated with total hepatic cholesterol (*P* < 0.05). Plasma from DKO, ApoE KO and het mice had the same efflux capacity. In the absence of CYP27A1, CAV‐1 overexpression might have an additional athero‐protective role by partly overcoming the defect in CYP27A1‐mediated cholesterol efflux.

**Abbreviations**
27‐OHC: 27‐hydroxycholesterol
ABCA1: ATP‐binding cassette A1
ABCG1: ATP‐binding cassette G1
ACTB: actin
ApoE: apolipoprotein E
BA: bile acid
CAV‐1: caveolin 1
CYP27A1: sterol 27‐hydroxylase
CYP3A11: cytochrome P450 3A11
CYP7A1: cytochrome P450 enzyme 7α‐hydroxylase
DKO: *Cyp27a1* KO/*ApoE* KO mice
eNOS: endothelial nitric oxide synthase
FXR: farnesoid X receptor
GC‐MS: gas chromatography–mass spectrometry
H&E: haematoxylin and eosin
HDL‐C: high‐density lipoprotein‐cholesterol
Het: *Cyp27a1* heterozygote/*ApoE* KO mice
HMGR: 3‐hydroxy‐3‐methylglutaryl‐CoA reductase
IHC: immunohistochemistry
IL: interleukin
KO: knockout
LDL‐C: low‐density lipoprotein‐cholesterol
LDLR: low‐density lipoprotein receptor
LXR: liver X receptor
NF: nuclear factor
NRF2: nuclear factor erythroid 2‐related factor 2
RCT: reverse cholesterol transport
SD: standard deviation
SHP: small heterodimer partner
SR‐B1: scavenger receptor B1
TG: triglyceride
TNFα: tumour necrosis factor α
WB: western blot
WD: western diet

Sterol 27‐hydroxylase (CYP27A1), a mitochondrial enzyme of the cytochrome P450 family, catalyses the hydroxylation of cholesterol at C27 to form 27‐hydroxycholesterol (27‐OHC) and cholestenoic acid. CYP27A1 plays a major role in cholesterol homoeostasis by metabolizing cholesterol into bile acids (BA). 27‐OHC is an endogenous inhibitor of the rate‐limiting enzyme of cholesterol biosynthesis [HMGCoA reductase (HMGR)]. CYP27A1 is also involved in cholesterol efflux 1, the first and rate‐limiting step in reverse cholesterol transport (RCT). The process of RCT channels cholesterol from extrahepatic tissues, including vessel walls, to the liver and subsequently eliminates it by conversion into BA. Through removal of excess cholesterol from the arterial wall, RCT may prevent the development of atherosclerosis 2. In previous *in vitro* studies, our group has demonstrated that CYP27A1 is directly involved in cholesterol efflux 3, 4.

To study the athero‐protective role of CYP27A1 *in vivo*,* Cyp27a1* KO mice were crossed with *ApoE* KO mice known for their propensity to spontaneously develop atherosclerosis, and the resulting offspring were fed a western diet (WD) for 3 and 6 months 5. The atherosclerosis observed in *ApoE* KO was abolished in *Cyp27a1/ApoE* double knockout (DKO) mice. DKO mice had hepatomegaly, elevated plasma HDL‐Cholesterol (HDL‐C), reduced cholesterol absorption and enhanced cholesterol elimination via increased *Cyp7a1* and *Cyp3a11* mRNA expression. The ATP‐binding cassette transporter ABCA1 (ABC‐A1) and scavenger receptor B1 (SR‐B1) implicated in cholesterol efflux were unaffected.

Caveolin‐1 (CAV‐1) is the major constituent of caveolae, which are 50–100 nm flask‐shaped invaginations of the plasma membrane present in most mammalian cells. It has been suggested that CAV‐1 is intimately involved in cholesterol trafficking 6. *In vitro,* studies have confirmed the role of CAV‐1 in RCT, since overexpression of *Cav‐1* in HepG2 cells increases the formation of caveolae and enhances cholesterol efflux 7. Furthermore, CAV‐1 has an additional athero‐protective role, as overexpression of *Cav‐1 in vivo* in the liver of C57BL/6J mice injected with adenoviruses encoding *Cav‐1* leads to increased plasma HDL‐C 8.

The aim of the study was to analyse CAV‐1 localization and expression in DKO mice and to investigate the effect of CYP27A1 downregulation on CAV‐1 expression in liver, aorta and macrophages and the ability of plasma to act as acceptor in a cholesterol efflux system. Due to the known association of CAV‐1 with liver lipid metabolism, we hypothesized that increased liver CAV‐1 expression would lead to increased triglyceride accumulation, increased lipogenesis and low‐density lipoprotein (LDL) internalization.

Thus, increased hepatic CAV‐1 could be considered as an additional athero‐protective mechanism, compensating for the defect in cholesterol efflux in DKO mice, in which *Cyp27a1* is not expressed.

## Experimental procedures

### Materials

Haematoxylin Gill no. 3 (GHS316), eosin Y aqueous (HT110216) and concanavalin A were from Sigma‐Aldrich (Buch, Switzerland). Cell culture medium was from Gibco (Lucerne, Switzerland). Primers were from Microsynth (Balgach, Switzerland), probes from Roche Diagnostics (Rotkreuz, Switzerland) and TaqMan assays from Life Technologies‐Applied Biosystems (Lucerne, Switzerland).

### Animals

Animal experimentation was approved by the Ethics Committee for Animal Experiments of the Veterinary Administration of the Canton of Berne, Switzerland, and conformed to the rules of the Swiss Federal Act on Animal Protection for the Care and Use of Laboratory Animals.

*Cyp27a1* ^(+/+)^/*ApoE* ^(−/−)^ (ApoE KO), *Cyp27a1* ^(+/−)^/*ApoE* ^(−/−)^ (het) and *Cyp27a1* ^(−/−)^/*ApoE* ^(−/−)^ (DKO) mice were bred/genotyped as previously published 5. Mice were maintained under a standard 12 : 12‐h light–dark cycle and fed *ad libitum*. At the age of 6 weeks, males were fed a WD for 2 months, containing 21% fat and 0.15% cholesterol (D‐12079B; Provimi Kliba AG, Kaiseraugst, Switzerland). Animals were fasted for 4 h before euthanasia by pentobarbital injection (300 mg·kg^−1^, Esconarkon, Streuli Pharma AG, Uznach, Switzerland). Animals were weighed, blood was collected into a tube containing 20–50 U heparin; livers and other organs were removed, washed with PBS, weighed and fixed in formalin at 4 °C or frozen in liquid nitrogen and stored at −70 °C. Heart and aorta were dissected, fixed in 4% paraformaldehyde for 24 h, transferred to PBS and stored at 4 °C.

### Isolation of peritoneal macrophages

In a second cohort of mice fed for 1 month with WD, peritoneal macrophages were collected 4 days following injection of concanavalin A (1.6 mg·kg^−1^ intraperitoneal). Cells were harvested in PBS and grown in macrophage serum‐free medium supplemented with 100 μg·mL^−1^ penicillin–streptomycin, 1% l‐glutamine, 1% sodium pyruvate 100 mm, 10% FBS, and 10 ng·mL^−1^ recombinant human macrophage colony‐stimulating factor (PeproTech, Rocky Hill, NJ, USA). Cells were incubated for 3 h at 5% CO_2_ and 100% humidity to allow specific macrophage adhesion. Adherent cells were washed three times with PBS and used for mRNA isolation.

### Characterization of atherosclerotic lesions

Lesions from aortas were stained with haemotoxylin and eosin (H&E) and quantified, as previously described 5.

### Biochemical analyses

Plasma cholesterol (TC), triglycerides (TG), LDL‐Cholesterol (LDL‐C) and high‐density lipoprotein‐Cholesterol (HDL‐C) were quantified using commercial kits (Wako Chemicals GmbH, Neuss, Germany). In liver homogenates, TG were measured using a kit (BioVision, Mountain View, CA, USA) as well as TC, cholesterol and cholesterol esters (Calbiochem‐Merck Millipore, Zug, Switzerland). All assays were conducted blind to group and in duplicate; the inter‐ and intracoefficient of variances were less than 10% and 5% respectively. Total BA were analysed in liver homogenates and faeces by gaschromatography–mass spectrometry (GC‐MS) using our previously described method 5.

### Cholesterol absorption by faecal dual‐isotope method

In a third cohort of mice fed WD, gavage was performed with 100 μL soybean oil containing 0.1 μCi [4‐^14^C] cholesterol and 0.2 μCi [22,23‐^3^H] β‐sitosterol. After gavage, each mouse was individually housed in a cage covered with Whatman paper and had free access to food and water. Faeces were collected daily for 3 days. Samples were extracted by saponification with 5 m KOH in 50% ethanol and heated at 80 °C for 1 h. Lipids were extracted by Folch method. The extracted lipids were transferred into scintillation vials and isotopes were counted in β‐counter. The percentage of cholesterol absorption was calculated as follows:

$$\frac{\frac{14C}{3H}\text{dose ratio}-\frac{14C}{3H}\text{faeces ratio}}{\frac{14C}{3H}\text{dose ratio}}\times 100.$$

### RNA extraction and real‐time PCR

RNA extraction and real‐time PCR in liver homogenates and isolated macrophages were conducted according to standard procedures previously described 5. Real‐time PCR was performed in triplicate with 100 ng cDNA/reaction. Primers and probes are those reported in our previous study 5 or from the Universal Probe library (Roche). β*‐Actin* (AM1720) was used as the internal standard. Quantification was performed by the relative quantification method using ApoE KO as calibrator.

### Immunohistochemistry (IHC)

Immunohistochemical staining was performed as previously described 5, 9. Slides were incubated with primary antibody (Anti‐CAV‐1‐ Santa Cruz Biotechnology, INC, Heidelberg, Germany) ([c‐894)] diluted for liver: 1 : 200 and aorta: 1 : 1500 in 1% BSA in PBS overnight at 4 °C. Slides were subsequently incubated with goat anti‐rabbit antibody (1 : 200; Santa Cruz Biotechnology, INC [sc‐2004]), followed by DAB chromogen (Dako, Hamburg, Germany) and counterstaining with haematoxylin. A negative control was performed by incubation without primary antibody.

All slides were blinded to group and assessed by the same observer (YTM). For analysis of sections, digital images of five randomly selected, high‐power (× 400 magnification) fields were captured on NIS‐Elements F2.20 microscope (Nikon Ltd, Kingston Upon Thames, Surrey, UK). Quantification of the specific staining was performed using the positive pixel algorithm of aperio image scope software 10. Accurate discrimination of immunolabelled regions was confirmed visually.

### Western blotting (WB)

Protein extraction using ~ 100 mg of powdered liver was performed in RIPA buffer containing protease and phosphatase inhibitor cocktail (10 μL·mL^−1^; Sigma‐Aldrich). For immunodetection, CAV‐1 (1 : 600 in TBST with 5% nonfat dry milk powder and 2% BSA) and horseradish peroxidase‐conjugated anti‐rabbit (1 : 10 000; Santa Cruz Biotechnology, INC [sc‐2004]) antibodies were used. Proteins were visualized by chemiluminescence agent using ECL Detection Reagents (GE Healthcare, Little Chalfont, UK). For internal standard, β‐actin [1 : 10 000 Santa Cruz Biotechnology, INC (sc‐69879)] was used. Densitometry of exposed films was performed using imagej software and the ratio of CAV‐1/β‐actin protein was expressed in relative light units.

### Cholesterol efflux

Cholesterol efflux was performed *in vitro* in RAW264.7 cells using 2% of various mice plasma for 2 h 3, 4, 7. Cholesterol efflux was calculated as percentage of labelled cholesterol released to the medium divided by the amount of total cholesterol in the medium and cells in each well. The assay was performed in quadruplicates and assay variability was controlled with a sample of pooled plasma.

### Statistical analysis

All tests were performed using the statistical package for social sciences (spss) for Windows version 22.0 (IBM Switzerland Ltd, Zürich, Switzerland) and graphpad prism version 6 (La Jolla, CA, USA). Summary data are presented as mean ± standard deviation (SD). One‐way ANOVA followed by either Tukey's or Gabriel's *post hoc* tests were used after normality was confirmed using the Kolmogorov–Smirnov and Shapiro–Wilk tests. Correlations between the parameters were tested with a Pearson's Rank correlation tests. The null hypothesis was rejected when *P* < 0.05.

## Results

### Effect of Cyp27a1 gene dosage on atherosclerotic phenotype at an early stage of atherosclerosis

ApoE KO, het and DKO mice were fed WD for 2 months; body and organ weights, plasma and hepatic lipids, hepatic BA and atherosclerotic plaque were analysed (Table 1).

**Table 1:** Effect of CYP27A1 expression on body and organ weight, plasma and liver lipids composition, intestinal cholesterol absorption and atherosclerosis in ApoE‐KO, het and DKO mice

Parameter	ApoE KO	het	DKO	*P*
	*n* = 6	*n* = 7	*n* = 6	
Weight				
Body (g)	27.7 ± 0.74	29.9 ± 1.62*	27.3 ± 1.12	**0.003**
Liver (g)	1.37 ± 0.21	1.66 ± 0.17	1.83 ± 0.40*	**0.03**
Spleen (g)	0.16 ± 0.03	0.16 ± 0.01	0.11 ± 0.02*	**0.01**
Lung (g)	0.17 ± 0.01	0.16 ± 0.02	0.16 ± 0.02	NS
Kidney (g)	0.16 ± 0.04	0.17 ± 0.01	0.17 ± 0.02	NS
Brain (g)	0.45 ± 0.02	0.45 ± 0.02	0.47 ± 0.01	NS
Plasma				
TC (mmol·L^−1^)	18.5 ± 4.94	18.4 ± 2.59	8.8 ± 1.26***	**< 0.0001**
LDL‐C (mmol·L^−1^)	12.9 ± 5.76	13.2 ± 5.35	7.8 ± 1.91	**0.1**
HDL‐C (mmol·L^−1^)	0.50 ± 0.13	0.51 ± 0.12	0.81 ± 0.07***	**0.0002**
TG (mmol·L^−1^)	1.17 ± 0.34	0.96 ± 0.27	1.93 ± 0.66*	**0.004**
Liver				
TC (μg·mg^−1^)	10.7 ± 1.44	9.5 ± 1.52	6.7 ± 1.34***	**0.0005**
Cholesterol (μg·mg^−1^)	9.1 ± 0.87	7.5 ± 1.04*	5.3 ± 0.68***	**< 0.0001**
Cholesterol ester (μg·mg^−1^)	1.60 ± 0.78	1.97 ± 0.84	1.31 ± 0.77	**0.36**
TG (nmol·mg^−1^)	42.2 ± 3.64	32.8 ± 11.0	67.9 ± 19.68*	**0.002**
BA (ng·g^−1^ liver)	13 996 ± 8646	7922 ± 4965	3026 ± 1250**	**0.002**
Faeces				
Cholesterol (DPM)	111 312 ± 20 788	120 409 ± 26 383	218 516 ± 51 930***	**< 0.0001**
Cholesterol absorption (%)	55 ± 4	57 ± 10	18 ± 6.93***	**< 0.0001**
BA (μg/24 h)	238.2 ± 51.3	250.5 ± 96.6	102 ± 18.9	**0.0019**
Aorta				
Lesion surface (mm^2^)	0.061 ± 0.034	0.045 ± 0.026	0.003 ± 0.005296**	**0.0024**

Results are presented as means ± SD. *P < 0.05, **P < 0.01, ***P < 0.001 versus ApoE‐KO.

Statistically significant values are represented in bold.

Het mice were heavier than their littermates (*P* = 0.003; Table 1). Liver weights and liver/body weight ratios were increased, whereas spleen weights decreased in DKO mice when compared to ApoE KO (*P* < 0.05 for all). The weights of kidneys, lungs and brains did not differ among the genotypes (Table 1).

Plasma composition analysis revealed that DKO had a twofold lower plasma TC concentration than both ApoE KO and het mice (*P* < 0.0001). In DKO mice, HDL‐C and TG concentrations increased by 60% and 65%, respectively, whereas LDL‐C concentration tended to decrease (Table 1).

In the liver, TC concentration was decreased by ~ 60% in DKO mice (*P* = 0.0005). The decrease was due to cholesterol and not cholesterol ester. Hepatic TG concentration increased by 60% in DKO mice (*P* = 0.002). Hepatic BA were significantly reduced in DKO mice compared to both ApoE KO and het mice (*P* = 0.0002; Table 1).

Cholesterol absorption was measured by faecal dual‐isotope incorporation. Following gavage with [4‐^14^C] cholesterol and [22,23‐^3^H] β‐sitosterol, DKO mice excreted significantly more faecal [4‐^14^C] cholesterol than their ApoE KO littermates (*P* < 0.0001, Table 1). Cholesterol absorption was threefold lower in DKO mice (*P* < 0.0001, Table 1), which also excreted significantly less BA than their ApoE KO littermates (Table 1).

In the aorta, there was no atherosclerosis in DKO mice (Table 1). The presence of cholesterol clefts and fibrosis were observed only in het and ApoE KO mice (data not shown).

### CAV‐1 expression in aorta and heart

**Figure 1:**
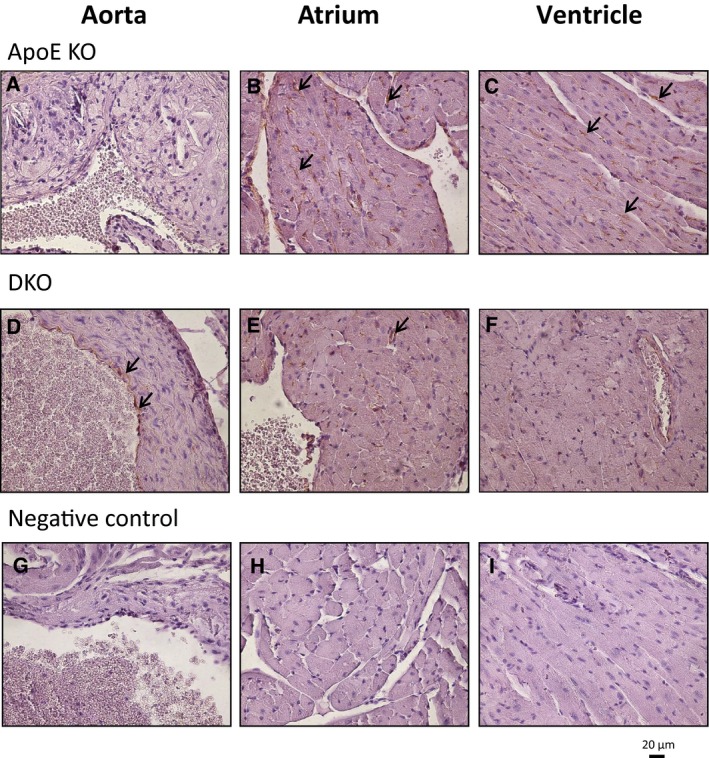
CAV‐1 protein expression in mice aorta and heart with and without atherosclerotic plaques. Aorta (A, D, G), atrium (B, E, H) and ventricles (C, F, I) sections were stained for CAV‐1 by IHC. (A) Almost no positive staining was observed in vascular cells making up plaques. In ApoE KO mice, CAV‐1 expression, which is localized around vessels in both the (B) atrium and (C) ventricles (black arrows), is increased. In DKO mice, higher CAV‐1 expression was seen in (D) endothelial cells (black arrows), with low expression in both (E) atrium and (F) ventricles. (G–I) are negative controls; 400× magnification.

Very little or no CAV‐1 protein expression was observed in vascular cells making up the plaques in ApoE KO mice (Fig. 1A). In addition, in areas where plaques were present, increased CAV‐1 was localized around vessels in both the atrium (Fig. 1B) and ventricles (Fig. 1C). Conversely, in DKO aortas without plaques, CAV‐1 was observed in endothelial cells (Fig. 1D), and only low expression was detected in the atrium (Fig. 1E) and ventricles (Fig. 1F).

### CAV‐1 expression in liver

**Figure 2:**
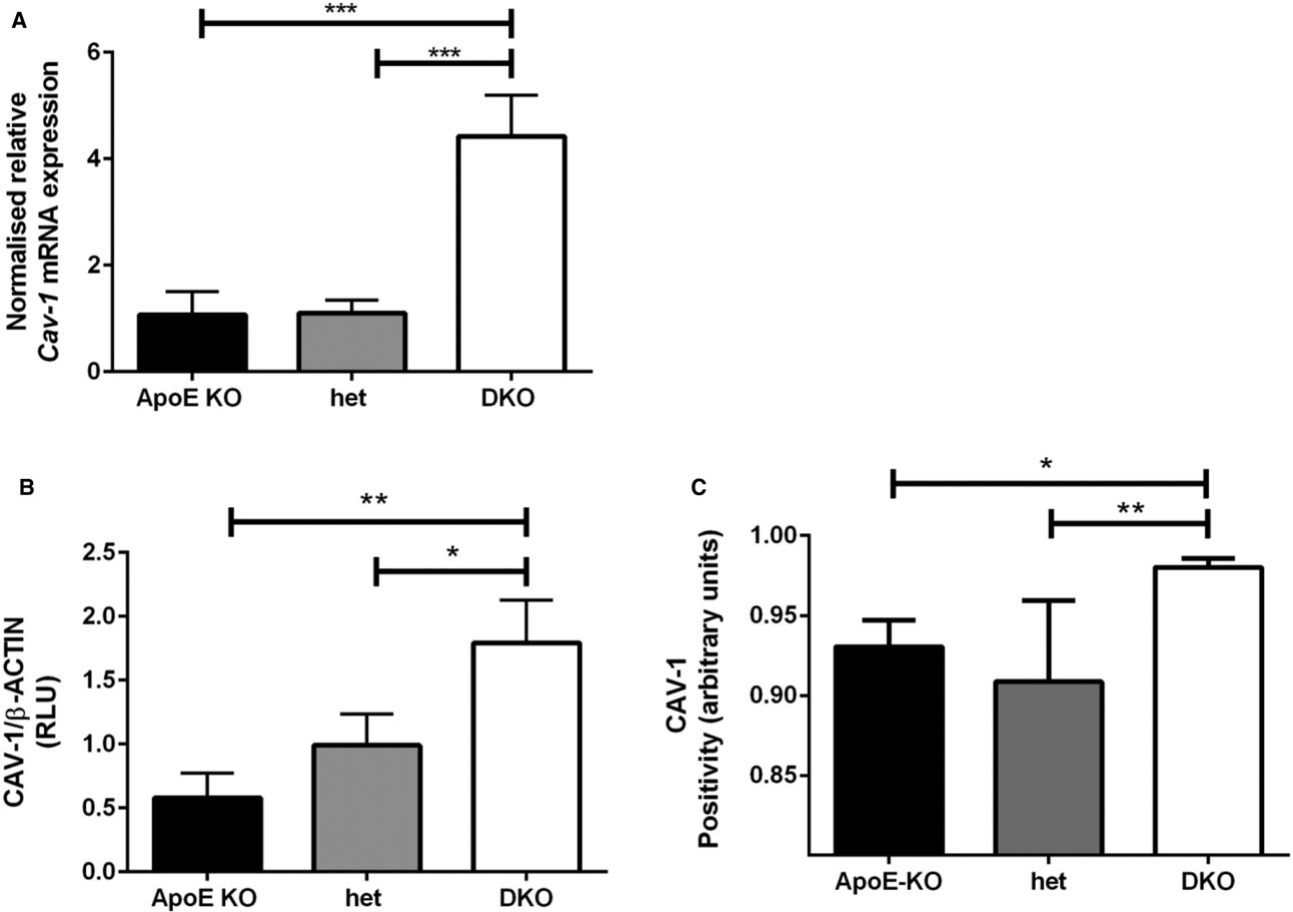
Genotype‐induced effect on expression of CAV‐1 in the liver. (A) *Cav‐1* mRNA expression was quantified by real‐time PCR, using β *Actin* as internal standard. CAV‐1 protein was quantified by WB (B) and IHC (C). Data are shown as mean ± SD. For statistical difference, *P < 0.05, **P < 0.01, ***P < 0.001.

*Cav‐1* mRNA expression was significantly increased in DKO mice liver (mean ± SD: 4.42 ± 0.78) compared to both ApoE KO (1.07 ± 0.44; *P* < 0.01) and het (1.13 ± 0.25; *P* < 0.05) mice (Fig. 2A).

**Figure 3:**
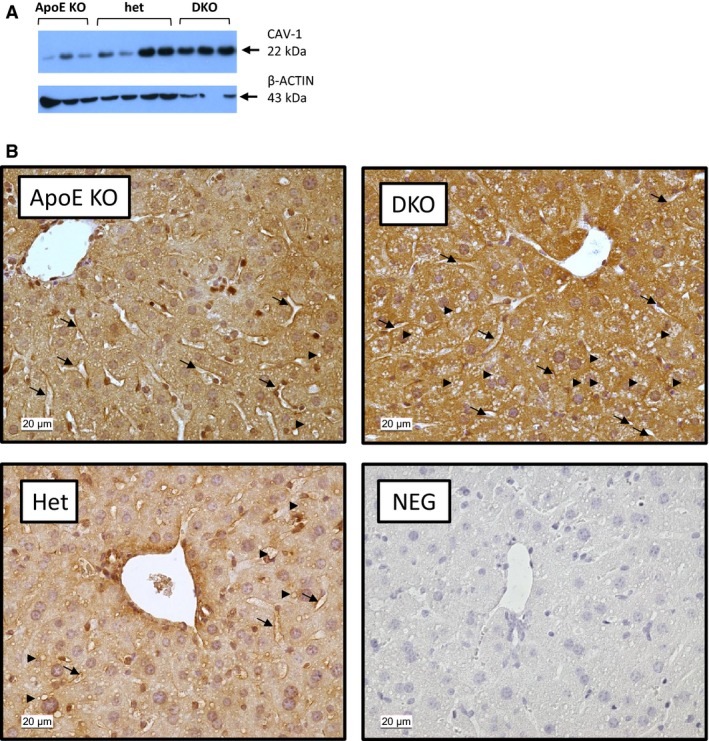
Genotype‐induced effect on expression of CAV‐1 in the liver. Representative WB (A) and IHC (B) of CAV‐1 in liver. CAV‐1 was detected as a 22‐KDa protein. The IHC staining localized to sinusoids (black arrows) and around micro/macrovesicular steatosis (black arrowheads); 400× magnification; scale bar = 20 μm.

The amount of CAV‐1 protein quantified by WB with a molecular weight of 22 kDa was about threefold higher in DKO (1.79 ± 0.33) and twofold higher in het (0.99 ± 0.24) than in ApoE KO (0.58 ± 0.19; *P* < 0.05 for all; Figs 2B and 3A) mice. The increased amount of CAV‐1 was also confirmed by IHC (0.98 ± 0.01 in DKO versus 0.93 ± 0.02 in ApoE KO and 0.91 ± 0.05 in het, *P* < 0.05; Figs 2C and 3B). CAV‐1 was localized around the sinusoid and the micro/macrovesicles (Fig. 3B).

Effect of Cyp27a1 gene dosage on expression of genes involved in cholesterol and lipid homoeostasis, cholesterol efflux, inflammation and oxidative stress in the liver and cholesterol efflux in isolated macrophages.

All gene expression results are summarized in Table 2. In the liver, the most upregulated genes were *Cyp7a1* and *Cyp3a11,* two genes involved in BA synthesis and cholesterol metabolism. Genes involved in cholesterol homoeostasis ([HMGR; *Hmgcr*]*,* sterol regulatory element‐binding protein 2 [*Srebf2*]*,* LDL receptor [*Ldlr*] and the transcription factor peroxisome proliferator‐activated receptor gamma [PPAR γ; *Pparγ*]) were also increased in DKO mice (*P* < 0.05 for all). The lipogenesis and triglyceride accumulation markers (Acetyl CoA carboxylase [ACC; *Acc*]*,* fatty acid synthase [FAS; *Fasn*] and diacylglycerol acyltransferase 1 [DGAT; *Dgat1*]) were also increased in DKO mice (*P* < 0.01 for both). Small heterodimer partner (SHP; *Nr0b2*) was reduced in DKO mice (*P* < 0.05 versus het). Farnesoid X receptor (FXR; *Nr1 h4*) and sterol regulatory element‐binding transcription factor 1 (SREBP1c; *Srebf1*) did not change between groups (ANOVA *P* > 0.05 for all).

**Table 2:** Genotype effects on expression of genes involved in lipid metabolism, inflammation and oxidative stress in liver and on cholesterol efflux in liver and macrophages

Parameter	ApoE KO	het	DKO	*P*
	*n* = 6	*n* = 7	*n* = 6	
Cholesterol and lipid homoeostasis				
Liver				
*Cyp7a1*	1.15 ± 0.73	2.88 ± 1.03	9.15 ± 3.10***	**< 0.0001**
*Cyp3a11*	1.00 ± 0.08	0.93 ± 0.25	18.32 ± 0.60***	**< 0.0001**
*Hmgcr*	1.02 ± 0.24	1.27 ± 0.40	9.20 ± 2.36***	**< 0.0001**
*Srebf2*	1.09 ± 0.40	1.71 ± 0.76	4.65 ± 1.99**	**0.003**
*Ldlr*	1.02 ± 0.20	1.75 ± 0.86	2.90 ± 1.05**	**0.003**
*Ppar*γ	1.03 ± 0.24	0.70 ± 0.08	3.30 ± 1.01***	**< 0.0001**
*Nr1 h4*	1.00 ± 0.29	0.90 ± 0.31	1.08 ± 0.36	NS
*Nr0b2*	1.10 ± 0.73	1.40 ± 0.30	0.22 ± 0.09	**0.0203**
*Srebf1*	1.14 ± 0.64	2.21 ± 1.33	1.50 ± 0.88	NS
*Acc*	1.01 ± 0.18	0.79 ± 0.08	1.40 ± 0.20**	**< 0.0001**
*Fasn*	1.10 ± 0.50	0.98 ± 0.48	3.90 ± 0.88***	**< 0.0001**
*Dgat1*	1.01 ± 0.18	0.85 ± 0.15	1.29 ± 0.25	**0.0087**
Cholesterol efflux				
Liver				
*Cyp27a1*	0.80 ± 0.20	0.50 ± 0.26*	0.00 ± 0.00***	**< 0.0001**
*Nr1 h3*	1.01 ± 0.19	1.05 ± 0.12	1.05 ± 0.11	NS
*Abca1*	1.01 ± 0.23	1.02 ± 0.06	0.64 ± 0.05**	**0.01**
*Abcg1*	1.04 ± 0.19	0.85 ± 0.22	0.52 ± 0.12***	**0.0005**
*Scarb1*	0.91 ± 0.14	0.88 ± 0.21	0.67 ± 0.11	NS
Macrophages				
*Cyp27a1*	1.00 ± 0.39	0.95 ± 0.53	0.00 ± 0.00	**0.0015**
*Cav‐1*	1.13 ± 0.73	0.59 ± 0.39	0.54 ± 0.10	NS
*Nr1 h3*	1.04 ± 0.29	1.49 ± 0.52	1.49 ± 0.20	NS
*Abca1*	1.05 ± 0.36	1.16 ± 0.43	0.89 ± 0.28	NS
*Abcg1*	1.03 ± 0.25	1.30 ± 0.62	1.89 ± 0.28	NS
*Scarb1*	1.02 ± 0.21	1.10 ± 0.20	1.13 ± 0.24	NS
Inflammation and oxidative stress				
Liver				
*Tnfα*	1.10 ± 0.42	1.00 ± 0.28	0.55 ± 0.22*	**0.02**
*Il1b*	1.04 ± 0.28	1.31 ± 0.41	1.10 ± 0.66	NS
*Il6*	1.12 ± 0.63	1.25 ± 0.94	1.93 ± 0.83	NS
*Il10*	1.09 ± 0.42	1.13 ± 0.40	1.20 ± 0.27	NS
*Nfkb*	1.01 ± 0.19	0.84 ± 0.09	0.89 ± 0.23	NS
*Nrf2*	1.05 ± 0.30	0.75 ± 0.05	0.92 ± 0.19	NS
*Nos3*	1.01 ± 0.20	0.84 ± 0.07	0.81 ± 0.17	NS

Results are presented as means ± SD. *P < 0.05, **P < 0.01, ***P < 0.001 versus ApoE‐KO.

Statistically significant values are represented in bold.

For hepatic genes involved in cholesterol efflux, *Cyp27a1* was confirmed to be absent in DKO mice and reduced by 50% in het mice. After 2 months of WD, *Abca1* (gene for ATP‐binding cassette [ABC] A1)*, Abcg1* (gene for ABCG1) and *Scarb1* (gene for SR‐B1) were reduced; *Nr1 h3* (gene for Liver X receptor α) remained unchanged in DKO mice.

Among the genes involved in inflammation in the liver, only tumour necrosis factor α (*Tnfα*) expression was lower in DKO mice compared to ApoE KO mice (*P* = 0.02). All other inflammatory genes (*Il1b* for IL‐1β, *Il6* for IL‐6, *Il10* for IL‐10, *Nfkb* for nuclear factor kappa B) and oxidative stress genes (*Nrf2* for nuclear factor erythroid 2‐related factor 2 [NRF2] and *Nos3* for endothelial nitric oxide synthase [eNOS]) were not different between groups (ANOVA *P* > 0.05 for all).

In isolated macrophages, the expression of all genes involved in cholesterol efflux was unchanged, except, as expected, *Cyp27a1* (ANOVA *P* = 0.0015, Table 2).

### Cholesterol efflux

The ability of plasma obtained from mice of all three genotypes to efflux cholesterol was tested in RAW 264.7 cells. The percentage of cholesterol efflux was slightly, but not significantly, higher when plasma from DKO were used (5.9 ± 1.5 versus 5.0 ± 0.9 in ApoE KO and 5.2 ± 0.5 in het; mean ± SD in %).

### Correlation analyses

*Cav‐1* mRNA expression positively correlated with both CAV‐1 protein expression data (WB: *R* ^2^ = 0.317; *r* = 0.563; *P* = 0.02; IHC: *R* ^2^ = 0.283; *r* = 0.532; *P* = 0.03), as did the WB and IHC results (*R* ^2^ = 0.232; *r* = 0.482; *P* = 0.04).

Both liver *Cav‐1* mRNA and CAV‐1 protein, measured by WB, negatively correlated with atherosclerotic plaques in all mice overall (mRNA: *R* ^2^ = 0.477; *r* = −0.691; protein: *R* ^2^ = 0.423; *r* = −0.655; *P* < 0.005 for both). Total liver cholesterol concentrations also negatively correlated with liver *Cav‐1* mRNA and both protein expression measurements (mRNA: *R* ^2^ = 0.47; *r* = −0.74; protein (WB): *R* ^2^ = 0.26; *r* = −0.51; protein (IHC): *R* ^2^ = 0.27; *r* = −0.52; *P* < 0.05 for all).

Finally in DKO mice, there was positive associations between *Cav‐1* with *Scarb1, Ldlr* and *Pparγ* (*Scarb1*:* r* = 0.80; *R* ^2^ = 0.65; *P* = 0.048; *Ldlr*:* r* = 0.82; *R* ^2^ = 0.55; *P* = 0.046; *Pparγ*:* r* = 0.82; *R* ^2^ = 0.68; *P* < 0.0001).

## Discussion

This study confirms our previous results observed in older DKO animals, highlighting the early age of atherosclerosis in ApoE KO and het mouse models^5^. This is also the first report on the effect of CYP27A1 suppression on CAV‐1 expression *in vivo* in DKO mice. These results suggest that increased CAV‐1 expression in the liver and in aortic endothelial cells is an additional protective mechanism against atherosclerosis development in DKO mice fed a WD.

The increased gene and protein expression of hepatic CAV‐1 in DKO mice initially suggested a contribution to eliminate cholesterol excess from the liver, as seen by decreased total liver/free cholesterol and the negative association between total liver cholesterol and *Cav‐1* mRNA/protein expression. In addition, as shown by our reported changes in intestinal cholesterol absorption, the absence of CYP27A1 expression in the intestine seems to be largely contributing to the antiatherosclerotic phenotype observed in our DKO mice.

Increased CAV‐1 might also compensate for the defect of CYP27A1‐mediated cholesterol efflux. Among the different cholesterol efflux mechanisms, the CAV‐1‐dependent pathway involves the transfer of cholesterol from intracellular compartments to caveolae for efflux 11. Cholesterol transport to the cell surface in cells expressing CAV‐1 is much faster than in the same cells lacking CAV‐1 11; in cells overexpressing CAV‐1, cholesterol efflux is increased 7. In our study, the ability of plasma to act as an acceptor in a cholesterol efflux system was not changed when DKO plasma was used. This indicates that the decreased CYP27A1 expression was compensated by other pathways of cholesterol efflux. Since *Abca1*,* Abcg1* and *Scarb1* were rather decreased, we conclude that the CAV‐1‐mediated pathway was enhanced, in line with the increased staining of CAV‐1 in endothelial cells of aortas from DKO mice. Second, as plasma acceptor capacity in DKO mice is not changed, the reduced atherosclerotic plaque formation in DKO mice is mainly due to lower plasma cholesterol, attributed to reduced intestinal absorption. Interestingly, the absence of *Cyp27a1* in macrophages was not compensated by the overexpression of other genes involved in cholesterol efflux. This suggests that, taken together, intestinal and hepatic changes account for the antiatherosclerotic lipid status in these DKO mice.

Caveolin 1 has previously been reported to be associated with the LDLR and the process of influx of LDL‐C from the circulation to the liver 12. Our data in DKO mice showing a strong positive correlation between hepatic *Cav‐1* and *Lldr* mRNA expression supports this. We can thus speculate that a cholesterol efflux‐independent mechanism takes place to reduce circulating LDL‐C and sequester it into the liver for subsequent degradation, as suggested by others 13.

**Figure 4:**
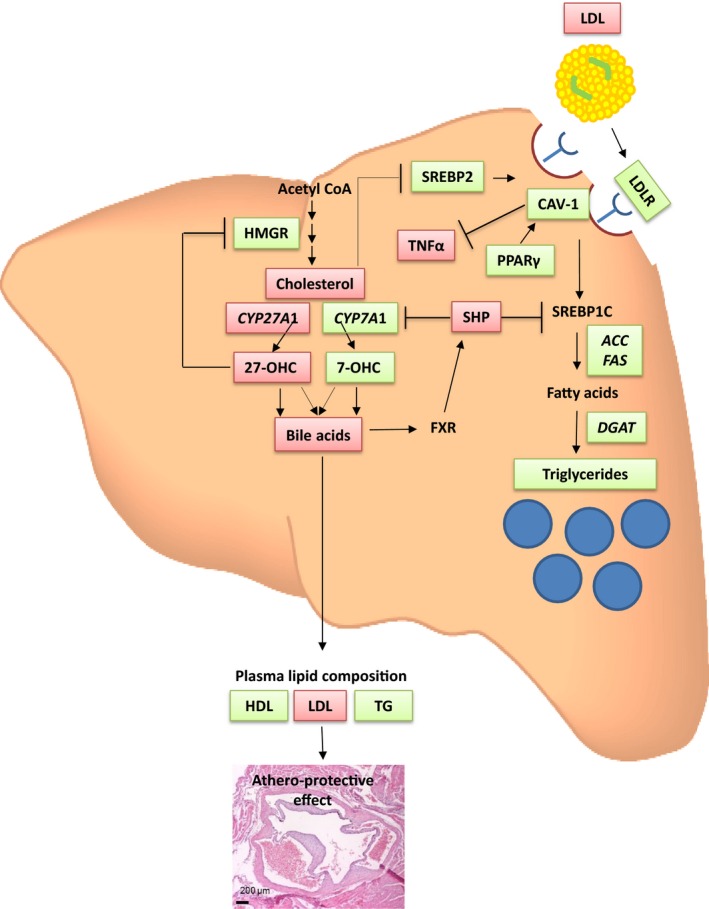
Beneficial effects of hepatic CAV‐1 on atherosclerosis protection in DKO mice. Atherosclerosis is mainly driven by high circulating cholesterol concentration, low HDL‐C and high LDL‐C. In plasma of DKO mice fed WD, the low levels of LDL‐C can be attributed to increased SREBP2 in the liver, which increases LDLR expression and LDL‐C uptake. CAV‐1 is also associated with the LDLR and the process of influx of LDL from the circulation to the liver. In DKO mice, SHP is downregulated, suppressing the inhibitory effect on SREBP1c and CYP7A1 as well as increasing TG and BA biosynthesis. The increased hepatic CAV‐1 expression appears to provide additional beneficial effects in promoting hepatic TG accumulation via increased DGAT expression and increasing hepatic gene expression of lipogenic markers (ACC and FAS). *Pparγ* was also increased in DKO mice and correlated positively with *Cav‐1* mRNA expression, supporting its role in upregulating CAV‐1. The lower TNFα, in combination with higher CAV‐1 expression in the liver further supports the potential anti‐inflammatory role for CAV‐1. In the absence of 27‐OHC in DKO mice, the rate‐limiting enzyme of cholesterol biosynthesis, HMGR is suppressed and cholesterol production is increased. Cholesterol is still metabolized to BA via the classical pathway of BA synthesis initiated by CYP7A1. The reduced transhepatic BA flux in DKO mice results in lower circulating LDL‐C, higher HDL‐C and TG concentrations; this leads to reduced atherosclerotic plaque formation. Green and red boxes represent up‐ and downregulation respectively.

Hepatic CAV‐1 expression has significant effects on OxLDL metabolism and on lipoprotein receptor levels 14. This observation could explain why no differences were observed for *Scarb1* between the groups and also why *Ldlr* expression was increased in DKO mice, illustrating the protective mechanism of CAV‐1 (summarized in Fig. 4). Therefore, increased hepatic CAV‐1 in the DKO mice could also be beneficial for the efficient removal of circulating oxLDL, preventing atherosclerosis development.

Bile acid increases FXR activity and the repressive effects of SHP thus preventing TG build‐up 15. The reduced BA concentrations and lower hepatic *Nr0b2* (gene for SHP) expression observed in DKO mice could also contribute to the hepatic and circulating TG accumulation. Furthermore, BA are associated with plasma lipid composition and with lower trans‐hepatic BA flux resulting in lower LDL‐C, higher HDL‐C and TG concentrations 16, as is the case in our DKO mice (Fig. 4).

In our DKO mice, the increased hepatic CAV‐1 appears to provide additional beneficial effects in promoting TG accumulation in the liver and increasing the expression of genes involved in lipogenesis (Fig. 4). This might be an adaptive response to deal with both CYP27A1 suppression and a WD, thus restricting the accumulation of cytotoxic free fatty acid as has been suggested 17. Similarly, the beneficial function of CAV‐1 was further highlighted by a previous study on hypercholesterolaemic rabbits treated with antennapedia‐CAV‐1 peptide reporting markedly less fat accumulation in the liver 18.

Several studies have tended to investigate the rather complex role of CAV‐1 leading to evidence of pro‐ or antiatherogenic function depending on localization, degree of expression and stage of atherosclerosis development. In ApoE KO mice fed a WD and infected for 4 weeks with AdPPARγ, CAV‐1 expression was induced in aortic vascular endothelial cells, smooth muscle cells and macrophages, and atherosclerotic lesions were attenuated 19. These results are in line with our DKO mice in which *Pparγ* was increased and correlated positively with *Cav‐1* mRNA expression 19.

In contrast, *Cav‐1/ApoE* double KO mice, with a > 2‐fold increase in LDL‐C 20, were protected from atherosclerosis, despite hypercholesterolaemia, underlying the role of CAV‐1 in transcytosis of LDL‐C across endothelial cells. Pavlides *et al*. also reported a cell‐specific role for CAV‐1 21; its absence in macrophage was proatherogenic, whereas its absence in endothelial cells was athero‐protective. In our DKO mice, the main effect of *Cyp27a1* suppression is a change in plasma lipid composition consisting of increased HDL‐C and decreased LDL‐C, as well as hepatic histology and lipid composition. We conclude that changes in hepatic CAV‐1 expression have a stronger impact on the atherosclerotic phenotype and that those in endothelial cells compensate the defect in cholesterol efflux.

In addition, CAV‐1 also appears to be an important mediator in response to inflammatory stimuli within the endothelium 22. Elevated CAV‐1 protein expression in aortas of diet‐induced obese rats fed a WD correlated with a decreased eNOS activity 23. In our DKO mice with increased CAV‐1, *Nos3* expression tended to be reduced (Table 2). The decreased *Tnfα* gene expression further suggests CAV‐1 has an anti‐inflammatory role in the liver (Table 2, Fig. 4).

To conclude, the advancement in the understanding of CAV‐1 and its influence on lipid homoeostasis and atherosclerosis could be a new approach for the treatment of metabolic syndrome. Our proposed mechanism (Fig. 4) on the beneficial effects of increased hepatic CAV‐1 on protection against atherosclerosis in DKO mice remains to be verified in humans with reduced CYP27A1 activity. The role of CAV‐1 in the intestine still needs to be investigated, particularly in DKO mice following restoration of hepatic BA synthesis, by examining the correlation between alterations in lipid profiles, liver and intestinal expression of CAV‐1, and atherosclerotic plaque formation.

## Author contributions

LZ designed and performed experiments, analysed the data and wrote the first draft of the manuscript. YTM analysed the data. BR performed experiments. BV made critical revisions and approved final version. HM conceived and designed the experiments, performed part of the experiments, analysed the data and contributed to the writing of the manuscript. GE conceived and designed the experiments, analysed the data and contributed to the writing of the manuscript.

## References

[1] Bjorkhem I , Andersson O , Diczfalusy U , Sevastik B , Xiu RJ , Duan C and Lund E (1994) Atherosclerosis and sterol 27‐hydroxylase: evidence for a role of this enzyme in elimination of cholesterol from human macrophages. Proc Natl Acad Sci USA 91, 8592–8596. 8078928 10.1073/pnas.91.18.8592 PMC44652

[2] Khera AV and Rader DJ (2010) Future therapeutic directions in reverse cholesterol transport. Curr Atheroscler Rep 12, 73–81. 20425274 10.1007/s11883-009-0080-0 PMC3315100

[3] Escher G , Hoang A , Georges S , Tchoua U , El‐Osta A , Krozowski Z and Sviridov D (2005) Demethylation using the epigenetic modifier, 5‐azacytidine, increases the efficiency of transient transfection of macrophages. J Lipid Res 46, 356–365. 15520456 10.1194/jlr.D400014-JLR200

[4] Escher G , Krozowski Z , Croft KD and Sviridov D (2003) Expression of sterol 27‐hydroxylase (CYP27A1) enhances cholesterol efflux. J Biol Chem 278, 11015–11019. 12531903 10.1074/jbc.M212780200

[5] Zurkinden L , Solca C , Vogeli IA , Vogt B , Ackermann D , Erickson SK , Frey FJ , Sviridov D and Escher G (2014) Effect of Cyp27A1 gene dosage on atherosclerosis development in ApoE‐knockout mice. FASEB J 28, 1198–1209. 24327605 10.1096/fj.13-233791 PMC4046167

[6] Fielding CJ and Fielding PE (2001) Caveolae and intracellular trafficking of cholesterol. Adv Drug Deliv Rev 49, 251–264. 11551398 10.1016/s0169-409x(01)00140-5

[7] Fu Y , Hoang A , Escher G , Parton RG , Krozowski Z and Sviridov D (2004) Expression of caveolin‐1 enhances cholesterol efflux in hepatic cells. J Biol Chem 279, 14140–14146. 14729661 10.1074/jbc.M311061200

[8] Frank PG , Pedraza A , Cohen DE and Lisanti MP (2001) Adenovirus‐mediated expression of caveolin‐1 in mouse liver increases plasma high‐density lipoprotein levels. Biochemistry 40, 10892–10900. 11535066 10.1021/bi0106437

[9] Mistry HD , Kurlak LO , Williams PJ , Ramsay MM , Symonds ME and Broughton Pipkin F (2010) Differential expression and distribution of placental glutathione peroxidases 1, 3 and 4 in normal and preeclamptic pregnancy. Placenta 31, 401–408. 20303587 10.1016/j.placenta.2010.02.011

[10] Mistry HD , McCallum LA , Kurlak LO , Greenwood IA , Broughton Pipkin F and Tribe RM (2011) Novel expression and regulation of voltage‐dependent potassium channels in placentas from women with preeclampsia. Hypertension 58, 497–504. 21730298 10.1161/HYPERTENSIONAHA.111.173740

[11] Smart EJ , Ying Y , Donzell WC and Anderson RG (1996) A role for caveolin in transport of cholesterol from endoplasmic reticulum to plasma membrane. J Biol Chem 271, 29427–29435. 8910609 10.1074/jbc.271.46.29427

[12] Ness GC , Kohlruss N and Gertz KR (2003) Association of the low‐density lipoprotein receptor with caveolae in hamster and rat liver. Biochem Biophys Res Commun 303, 177–181. 12646183 10.1016/s0006-291x(03)00319-x

[13] Frank PG , Pavlides S , Cheung MW , Daumer K and Lisanti MP (2008) Role of caveolin‐1 in the regulation of lipoprotein metabolism. Am J Physiol Cell Physiol 295, C242–C248. 18508910 10.1152/ajpcell.00185.2008 PMC2493562

[14] Truong TQ , Brodeur MR , Falstrault L , Rhainds D and Brissette L (2009) Expression of caveolin‐1 in hepatic cells increases oxidized LDL uptake and preserves the expression of lipoprotein receptors. J Cell Biochem 108, 906–915. 19718657 10.1002/jcb.22321

[15] Watanabe M , Houten SM , Wang L , Moschetta A , Mangelsdorf DJ , Heyman RA , Moore DD and Auwerx J (2004) Bile acids lower triglyceride levels via a pathway involving FXR, SHP, and SREBP‐1c. J Clin Investig 113, 1408–1418. 15146238 10.1172/JCI21025 PMC406532

[16] Leiss O and von Bergmann K (1982) Different effects of chenodeoxycholic acid and ursodeoxycholic acid on serum lipoprotein concentrations in patients with radiolucent gallstones. Scand J Gastroenterol 17, 587–592. 7178821 10.3109/00365528209181063

[17] Simard JR , Meshulam T , Pillai BK , Kirber MT , Brunaldi K , Xu S , Pilch PF and Hamilton JA (2010) Caveolins sequester FA on the cytoplasmic leaflet of the plasma membrane, augment triglyceride formation, and protect cells from lipotoxicity. J Lipid Res 51, 914–922. 20388923 10.1194/jlr.M900251 PMC2853459

[18] Chen YH , Lin WW , Liu CS , Hsu LS , Lin YM and Su SL (2014) Caveolin‐1 provides palliation for adverse hepatic reactions in hypercholesterolemic rabbits. PLoS One 9, e71862. 24475013 10.1371/journal.pone.0071862 PMC3901645

[19] Hu Q , Zhang XJ , Liu CX , Wang XP and Zhang Y (2010) PPARgamma1‐induced caveolin‐1 enhances cholesterol efflux and attenuates atherosclerosis in apolipoprotein E‐deficient mice. J Vasc Res 47, 69–79. 19729954 10.1159/000235927

[20] Frank PG , Lee H , Park DS , Tandon NN , Scherer PE and Lisanti MP (2004) Genetic ablation of caveolin‐1 confers protection against atherosclerosis. Arterioscler Thromb Vasc Biol 24, 98–105. 14563650 10.1161/01.ATV.0000101182.89118.E5

[21] Pavlides S , Gutierrez‐Pajares JL , Katiyar S , Jasmin JF , Mercier I , Walters R , Pavlides C , Pestell RG , Lisanti MP and Frank PG (2014) Caveolin‐1 regulates the anti‐atherogenic properties of macrophages. Cell Tissue Res 358, 821–831. 25322709 10.1007/s00441-014-2008-4

[22] Shiroto T , Romero N , Sugiyama T , Sartoretto JL , Kalwa H , Yan Z , Shimokawa H and Michel T (2014) Caveolin‐1 is a critical determinant of autophagy, metabolic switching, and oxidative stress in vascular endothelium. PLoS One 9, e87871. 24498385 10.1371/journal.pone.0087871 PMC3912129

[23] Yang N , Ying C , Xu M , Zuo X , Ye X , Liu L , Nara Y and Sun X (2007) High‐fat diet up‐regulates caveolin‐1 expression in aorta of diet‐induced obese but not in diet‐resistant rats. Cardiovasc Res 76, 167–174. 17599814 10.1016/j.cardiores.2007.05.028

